# Methotrexate Reduces the Probability of Discontinuation of TNF Inhibitors in Seropositive Patients With Rheumatoid Arthritis. A Real-World Data Analysis

**DOI:** 10.3389/fmed.2021.692557

**Published:** 2021-06-29

**Authors:** Borja Hernández-Breijo, Claudia M. Brenis, Chamaida Plasencia-Rodríguez, Ana Martínez-Feito, Marta Novella-Navarro, Dora Pascual-Salcedo, Alejandro Balsa

**Affiliations:** ^1^Immuno-Rheumatology Research Group, Hospital La Paz Institute for Health Research-IdiPAZ, Madrid, Spain; ^2^Rheumatology, La Paz University Hospital, Madrid, Spain; ^3^Immunology, La Paz University Hospital, Madrid, Spain

**Keywords:** rheumatoid arthritis, TNF inhibitor, drug survival, methotrexate, seropositivity

## Abstract

Tumor necrosis factor inhibitors (TNFi) are widely used for the treatment of patients with rheumatoid arthritis (RA), however a considerable percentage of patients discontinued the therapy. The aim of this study is to explore real-world TNFi survival, stratified for seropositivity, and to determine the factors that may influence it. This is a retrospective, observational and longitudinal study, using real-world data of patients, who started their first TNFi therapy between 1999 and 2018 from the RA-PAZ cohort. Patients were considered seropositive if they showed positive serum levels of either RF, ACPA, or both. Treatment survival was analyzed using Kaplan-Meier curves, and Cox proportional hazards models were used to compare the risks of TNFi discontinuation for seronegative and seropositive patients. Of the included 250 patients, 213 (85%) were seropositive. Results showed that TNFi survival did not depend on seropositivity status. However, median survival time was significant longer for seropositive patients who received concomitant MTX compared to patients who did not receive it (median [95% CI]: 3.3 yr. [2.3–4.2] vs. 2.6 yr. [1.7–3.6], respectively; *p* = 0.008). Furthermore, seropositive patients who received concomitant MTX were 49% less likely to discontinue TNFi therapy than patients who did not receive it (HR: 0.51; 95% CI: 0.35–0.74). In addition, we found that in seropositive patients, the use of prednisone throughout the TNFi treatment was associated with a higher likelihood of therapy discontinuation (OR: 2.30; 95% CI: 1.01–5.23). In conclusion, these data provide evidence to support the use of concomitant MTX in seropositive patients to prolong the effectiveness and the survival of the TNFi therapy. Moreover, the co-administration of prednisone in seropositive patients receiving TNFi was highly associated with TNFi discontinuation.

## Introduction

Tumor necrosis factor inhibitors (TNFi) are widely used for the treatment of patients with rheumatoid arthritis (RA), who do not respond to conventional synthetic disease-modifying anti-rheumatic drugs (csDMARDs). However, 20–40% of patients do not achieve an adequate clinical response and stop or discontinue the TNFi therapy (1). The latest breakthroughs in the physiopathology of RA highlighted the activation of B cells as the trigger of the joint flare initiation, and, therefore, gave them a central role in the pathogenesis of the disease (2, 3). This joint inflammation triggered by B cells is partly mediated by soluble cytokines, such as IL6, APRIL, or BAFF, which promote B cell activation and differentiation into short-lived plasma cells, producing autoantibodies as rheumatoid factor (RF) or anti-citrullinated protein antibodies (ACPA) (4). Furthermore, serum autoantibodies have been correlated with a higher disease activity (5, 6), which has been associated with an increase of TNFi clearance due to the inflammatory sink that would lead to an early drug administration stopping (7, 8). In this line, some studies have pointed out that the autoantibody seropositive status, mainly due to RF or ACPA, may be influencing the survival of TNFi as well as blood TNFi levels (9, 10). One of the factors that prolongs TNFi treatment survival is the co-administration of csDMARDs, especially methotrexate (MTX). In fact, blood concentrations are higher for patients with RA treated with TNFi when MTX is co-prescribed from the start of treatment (11–13), and it is already recommended by the European Alliance of Associations for Rheumatology (EULAR) (14).

The main objective of this study is to investigate the effect of concomitant MTX use on the survival of TNFi in seronegative and seropositive patients with RA. In addition, the influence of the patients' characteristics on TNFi discontinuation will be analyzed according to the seropositivity status.

## Materials and Methods

### Patients

This was a retrospective, observational and longitudinal study, using real-world data from the RA-PAZ cohort (15). The study included data from 250 patients with RA who started TNFi (infliximab, adalimumab, etanercept, golimumab, or certolizumab) as first ever bDMARD between 1999.12.03 and 2018.09.11, according to national recommendations (16). All the included patients were adults (age over 18). Patients diagnosed before 2010 fulfilled the 1987 ACR classification criteria for RA (17), and patients diagnosed since 2010 fulfilled the ACR/EULAR 2010 classification criteria for RA (18). All of them had moderate or high disease activity (DAS283 > 0.2). Approval was obtained from the Institutional Ethics Committee from La Paz University Hospital (PI-1155) in accordance with the Helsinki Declaration. All patients signed an informed consent document before inclusion.

### Demographical and Clinical Data

Baseline demographical and clinical characteristics were collected [age, sex, disease duration, body mass index (BMI), smoking habit, Disease Activity Score 28 (DAS28), c-reactive protein (CRP), rheumatoid factor (RF), anti-citrullinated protein antibodies (ACPA)]. Moreover, the concomitant use of methotrexate (MTX) and prednisone was also registered. Information on causes for treatment discontinuation (lack of efficacy and others) was collected. In case of infliximab treatment, patients were followed-up at the baseline and at time of the infusion visit. For subcutaneous treatments (adalimumab, etanercept, golimumab, or certolizumab), patients were followed-up at the baseline, at 3 months, at 6 months and every 6 months thereafter throughout the TNFi treatment.

### Outcome

The primary outcome was persistence with first TNFi therapy, which was defined as the time that patients continued receiving TNFi treatment. Patients who discontinued treatment due to remission or loss of follow-up were not included in the study.

### Measurement of the Serum RF and ACPA

Serum samples were collected at time of diagnosis and prior TNFi initiation in order to perform other determinations as part of laboratory routine, and then were stored at −20°C. Serum RF and ACPA from patients who initiated TNFi at late 90's and early 2000's were determined *a posteriori*. Serum RF concentrations were measured by nephelometry using the BNII System and N Latex RF Kit (Siemens Healthcare Diagnostics *Inc*., Newark, USA) according to the instruction manual. RF > 20 IU/mL was considered positive. Serum ACPA was determined by ELISA as anti-CCP2 with the ImmunoscanCCPlus kit (Svar, Malmö, Sweden). ACPA > 25 IU/mL was considered positive. RF and ACPA seropositivities were confirmed at least in two determinations (at time of diagnosis and prior TNFi initiation).

### Statistical Analyses

The cohort was divided according to autoantibodies seropositivity. Patients were considered seropositive if they showed positive serum levels of either RF, ACPA or both. Descriptive analyses were performed for the demographic and clinical variables. The results are shown as mean and SD (or median and interquartile range [IQR]) for continuous variables, and absolute numbers and relative frequencies for categorical variables. The frequency data were compared using the Fisher's exact tests. Comparisons of unpaired continuous data were conducted using the unpaired *t*-test or Mann-Whitney *U*-test, depending on data distribution. Associations between demographic and clinical variables and TNFi discontinuation were evaluated using univariable and multivariable logistic regression models, and data were presented as odds ratios, OR and 95% confidence intervals, CI. Any variable having a *p* ≤ 0.1 at the univariable test was selected for the multivariable analysis.

Treatment survival was analyzed using Kaplan-Meier curves, and all groups were compared using the log-rank test. Cox proportional hazards models were used to compare the risk of TNFi discontinuation between seronegative and seropositive patients. Multivariable adjustment was performed for the following baseline covariates: age, sex, BMI, smoking habit, disease duration, baseline DAS28, baseline CRP, type of TNFi, cause of TNFi discontinuation and use of prednisone.

A *p* < 0.05 was considered as statistically significant. The Statistical Package for the Social Sciences version 24 (SPSS, Chicago, IL, USA) was used for the analyses. The GraphPad Prism version 7 (GraphPad Software, San Diego, CA, USA) was used to prepare the graphs.

## Results

### Patients' Characteristics

Demographic and clinical characteristics are shown in Table 1. One-hundred nine patients (44%) received infliximab, 58 (23%) adalimumab, 49 (19%) etanercept, 29 (12%) certolizumab and 5 (2%) golimumab. The mean baseline disease activity (DAS28) was 5.2. Two-hundred thirteen patients (85%) were seropositive and 37 (15%) were seronegative. Out of two-hundred fifteen patients (86%) who discontinued TNFi treatment, 185 (87%) were seropositive and 30 (81%), seronegative. Seropositive patients showed significant longer median disease duration (8.1 yr. vs. 4.0 yr.; *p* < 0.001) and higher mean baseline DAS28 (5.2 vs. 4.7; *p* < 0.05) than seronegative. These differences, between seropositive and seronegative groups, were mainly due to the comparison of patients receiving concomitant MTX (disease duration: 7.7 yr. in seropositive group vs. 3.5 yr. in seronegative group, *p* < 0.05; baseline DAS28: 5.3 in seropositive group vs. 4.6 in seronegative group; *p* < 0.05). In general, 215 patients discontinued TNFi (185 in seropositive group and 30 in seronegative group). Lack of efficacy was the main cause of TNFi discontinuation (118, 47%), happening after the first 6 months of TNFi treatment in most cases (107/118, 91%). In global, 70% of the patients received concomitant MTX (68% in seropositive group and 78% in seronegative group). Within the seropositive group, the proportion of TNFi discontinuation was similar between patients with or without MTX (87 vs. 86%, respectively). Nevertheless, the causes of discontinuation were different (*p* < 0.05). The seropositive patients taking MTX more frequently discontinued TNFi treatment due to the lack of efficacy vs. other causes (61 vs. 31%, respectively); however, seropositive patients not taking MTX, causes of discontinuation were more balanced between other causes and lack of efficacy (57 vs. 43%, respectively). Sixty-six percent of the patients used prednisone along the TNFi treatment. However, seronegative patients who were treated with concomitant MTX received significantly prednisone with more frequency (76 vs. 37%; *p* < 0.05) than patients who did not receive concomitant MTX.

**Table 1:** Patient characteristics.

**Patient characteristics**	**Pooled *n* = 250**	**Seropositive (85%)**			**Seronegative (15%)**		
		**Pooled**	**MTX**	**No MTX**	**Pooled**	**MTX**	**No MTX**
		***n* = 213**	***n* = 146 (68%)**	***n* = 67 (32%)**	***n* = 37**	***n* = 29 (78%)**	***n* = 8 (22%)**
Age (yr)	541 ± 4	541 ± 4	541 ± 4	561 ± 5	521 ± 7	521 ± 6	491 ± 9
Female	208 (83)	175 (82)	123 (84)	52 (78)	33 (89)	25 (86)	8 (100)
BMI (kg/m^2^) (*n* = 232)	25.6 (22.1–29.7)	25.4 (22.2–29.7)	25.4 (22.2–30.4)	25.2 (22.0–29.1)	26.5 (21.2–28.7)	26.5 (23.4–29.1)	22.1 (20.8–28.3)
BMI <30 (kg/m^2^)	179 (77)	152 (76)	100 (73)	52 (84)	27 (82)	21 (81)	6 (86)
BMI ≥ 30 (kg/m^2^)	53 (23)	47 (24)	37 (27)	10 (16)	6 (18)	5 (19)	1 (14)
**Smoking habit**							
Non-smokers	159 (63)	133 (62)	95 (65)	38 (57)	26 (70)	21 (73)	5 (62)
Ex-smokers	52 (21)	47 (22)	29 (20)	18 (27)	5 (13)	3 (10)	2 (25)
Smokers	39 (16)	33 (16)	22 (15)	11 (16)	6 (16)	5 (17)	1 (13)
RF positive	187 (75)	187 (88)	129 (88)	58 (87)	–	–	–
ACPA positive	193 (79)	193 (92)	135 (92)	58 (92)	–	–	–
Disease duration (yr)	7.8 (3.3–6.2)	8.1 (3.9–13.0)***	7.7 (3.5–12.9)^**¶**^	9.8 (4.2–14.5)	4.0 (1.6–10.9)	3.5 (1.6–10.9)	6.7 (1.5–10.3)
Baseline DAS28	5.21 ± 0.4	5.21 ± 0.4 *	5.31 ± 0.3^**¶**^	5.21 ± 0.5	4.71 ± 0.2	4.61 ± 0.4	4.90 ± 0.6
Baseline CRP (mg/dL)	6 (2.6–18.2)	6.4 (3.0–16.3)	5.7 (3.0–16.5)	8.3 (2.3–16.3)	3.1 (1.4–28.9)	3.0 (1.4–24.7)	8.7 (2.1–50.4)
**TNFi type**							
Infliximab	109 (44)	96 (45)	69 (47)	27 (40)	13 (35)	12 (41)	1 (12.5)
Adalimumab	58 (23)	48 (23)	32 (22)	16 (24)	10 (27)	8 (28)	2 (25)
Etanercept	49 (19)	38 (18)	25 (17)	13 (19)	11 (30)	7 (24)	4 (50)
Certolizumab	29 (12)	26 (12)	16 (11)	10 (15)	3 (8)	2 (7)	1 (12.5)
Golimumab	5 (2)	5 (2)	4 (3)	1 (2)	0	0	0
**Cause of discontinuation**							
Lack of efficacy	118 (47)	102 (48)	77 (53)^**#**^	25 (37)	16 (46)	12 (41.5)	4 (50)
Other causes	97 (39)	83 (39)	50 (34)	33 (50)	14 (35)	12 (41.5)	2 (25)
MTX use	175 (70)	146 (68)	–	–	29 (78)	–	–
MTX dose (mg/week)	15.0 (0.0–20.0)	15.0 (0.0–20.0)	15 (12.5–20.0)	–	15.0 (7.5–20.0)	15.0 (13.7–20.0)	–
Prednisone use	164 (66)	139 (65)	99 (68)	40 (60)	25 (68)	22 (76)^**#**^	3 (37)

The table shows mean ± SD, median (IQR) or absolute number (percentage) for patients included (n = 250). The results are stratified by seropositivity status. P < 0.05 was considered statistically significant. Significant statistical differences are indicated as: (*) comparison between seronegative and seropositive pooled patients; (#) comparison between patients according to the use of concomitant MTX, within each seroposivity status; (¶) comparison between seronegative and seropositive patients according to the use of concomitant MTX. P < 0.05 (*/#/¶), p < 0.01 (**/##/¶¶), p < 0.001 (***/###/¶¶¶).

*BMI, body mass index; DAS28, disease activity score-28; CRP, c-reactive protein; TNFi, tumor necrosis factor inhibitor; RF, rheumatoid factor; ACPA, anti-citrullinated peptide antibody; MTX, methotrexate*.

### Survival Analysis for the TNFi Treatment

**Figure 1:**
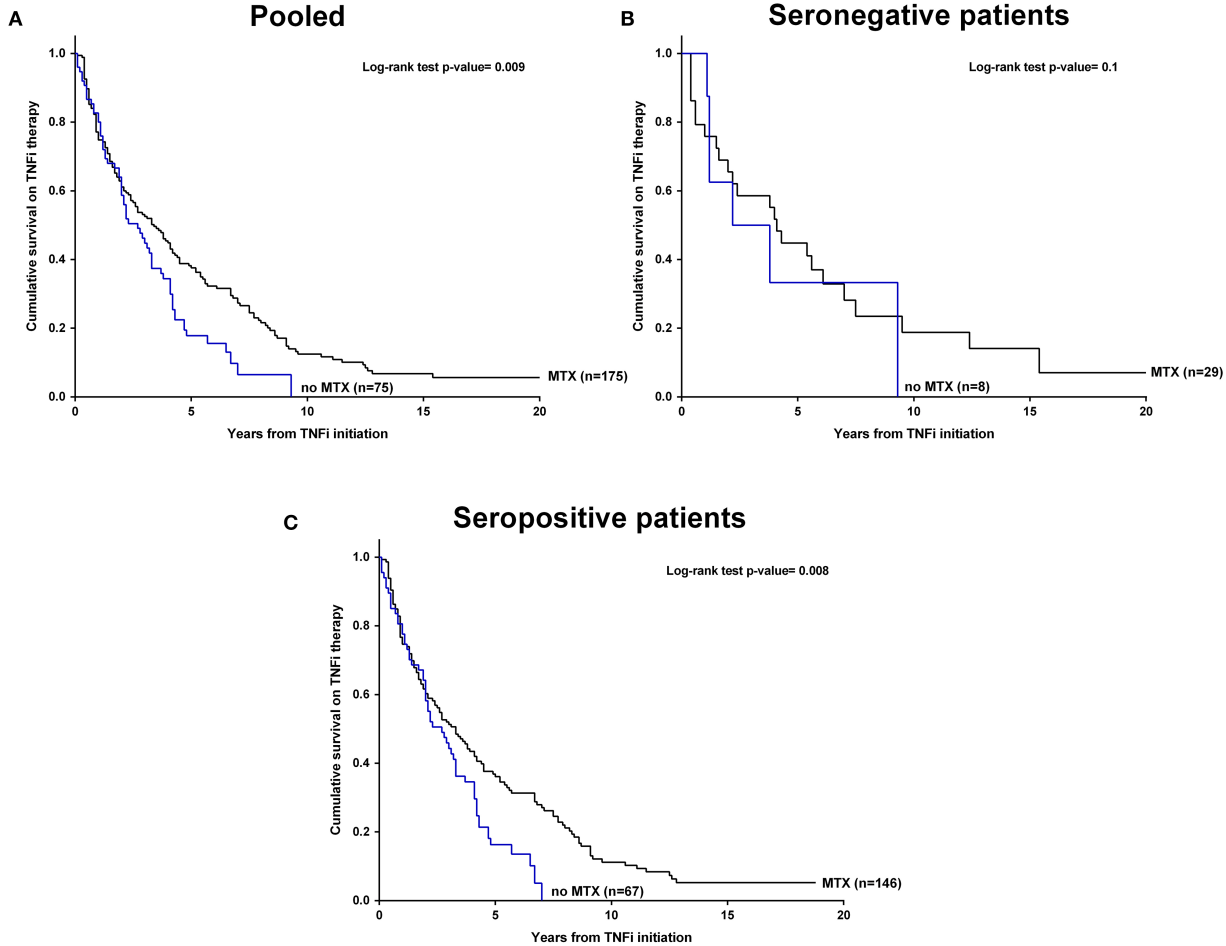
Kaplan-Meier curves of cumulative survival on TNFi therapy by concomitant use of MTX in **(A)** cohort patients, **(B)** seronegative patients, and **(C)** seropositive patients. *P* < 0.05 was considered statistically significant. MTX, methotrexate; TNFi, tumor necrosis factor inhibitors.

As it was described above, the overall frequency of TNFi discontinuation was 215 (86%) patients. The percentage of patients who discontinued TNFi during the follow-up was not significant different between the seropositivity groups (87% in seropositive patients *vs*. 81% in seronegative patients). According to the use of concomitant MTX, the frequencies of patients who discontinued TNFi were not statistically different in none of the seropositivity groups (Table 1). In order to analyze the persistence of TNFi therapy Kaplan-Meier curves were performed along with the median TNFi survival time in all patients as well as in both seropositivity groups, stratified using concomitant MTX (Figure 1). Overall, we found that patients who received concomitant MTX showed median longer survival time than patients who did not receive it (median [95% CI]: 3.3 yr [2.4–4.3] vs. median [95% CI]: 2.6 yr [1.8–3.5]) (*p* = 0.009). In the seropositive group, we found that the median TNFi survival time was significantly longer in patients who received concomitant MTX (median [95% CI]: 3.3 yr [2.3–4.2]) than in patients who did not received it (median [95% CI]: 2.6 yr [1.7–3.6]) (*p* = 0.008). Interestingly, this difference was not found for seronegative patients due to the fact that median TNFi survival time was statistically similar in patients who received concomitant MTX (median [95% CI]: 4.1 yr [3.1–5.1]) and in patients who did not received it (median [95% CI]: 2.2 yr [0.0–5.3]) (*p* = 0.7). In this regard, we perform an adjusted COX-regression analysis which concluded that seropositive patients who received concomitant MTX were 49% less likely to discontinue TNFi therapy than patients who did not receive it (HR: 0.51; 95% CI: 0.35–0.74; *p* = 0.0004). In addition, we found that in our cohort, the time (year) of TNFi initiation was crucial for the treatment survival. Seropositive patients who started the TNFi more recently were more likely to discontinue TNFi therapy earlier (HR: 1.84; 95% CI: 1.54–2.20; *p* < 0.0001) (Table 2). In addition, these results found for seropositive patients was similar than the obtained when all the patients was considered (Supplementary Table 2).

**Table 2:** COX proportional hazard estimates (95% CI) for TNFi therapy survival, stratified by seropositivity.

	**Seropositive**			**Seronegative**		
**Patient characteristics**	**HR**	**95% CI**	***p*-value**	**HR**	**95% CI**	***p*-value**
MTX use (unadjusted)	0.52	0.35–0.78	**0.001**	0.05	0.00–1.28	0.07
MTX use (adjusted)	0.51	0.35–0.74	**0.0004**	0.26	0.05–1.38	0.1
Time (year) of TNFi initiation (unadjusted)	1.85	1.54–2.23	** <0.0001**	0.92	0.34–2.50	0.9
Time (year) of TNFi initiation (adjusted)	1.84	1.54–2.20	** <0.0001**	0.92	0.34–2.50	0.9

*Analysis adjusted for age, sex, BMI, smoking habit, disease duration, baseline DAS28, baseline CRP, type of TNFi, cause of TNFi discontinuation, use of concomitant MTX, time of TNFi initiation, and use of prednisone. Significant statistical differences are noted in bold. P < 0.05 was considered statistically significant. BMI, body mass index; DAS28, disease activity score-28; CRP, c-reactive protein; MTX, methotrexate; TNFi, tumor necrosis factor inhibitor; HR, hazard ratio; CI, confidence interval*.

### Association Between Patient Characteristics and TNFi Discontinuation

First of all, we analyzed the association between patient characteristics and TNFi discontinuation. After the univariable logistic regression analyses, we found that the use of prednisone throughout the TNFi treatment (OR: 2.29; 95% CI: 1.11–4.71) was significant associated with TNFi discontinuation. The result remained associated after the multivariable analysis, although not significant (OR: 2.05; 95% CI: 0.97–4.32) (Supplementary Tables 3, 4). Then, patients were stratified by seropositivity status. Associations of the different demographic and clinical variables with the discontinuation of TNFi therapy were analyzed by univariable logistic regression analyses. We found that the use of prednisone throughout the TNFi treatment (OR: 2.46; 95% CI: 1.10–5.51) was significant associated with TNFi discontinuation only in seropositive patients. Moreover, having a higher baseline DAS28 (OR: 1.28; 95% CI: 0.96–1.71) and receiving a monoclonal TNFi (OR: 2.07; 95% CI: 0.83–5.12) tended to be associated with TNFi discontinuation. On the other hand, no significant associations among demographic and clinical variables with the discontinuation of TNFi therapy were found in the group of seronegative patients (Table 3). Then, a multivariable analysis including demographic and clinical variables with a *p* ≤ 0.1 in the univariable analysis (baseline DAS28, the type of TNFi received and the use of prednisone) was performed for seropositive patients. The results showed that only the use of prednisone throughout the TNFi treatment (OR: 2.30; 95% CI: 1.01–5.23) remained significantly associated with TNFi discontinuation in seropositive patients (Table 4).

**Table 3:** Association between patient characteristics and TNFi discontinuation (univariable analysis), stratified by seropositivity.

	**Seropositive**			**Seronegative**		
**Patient characteristics**	**OR**	**95% CI**	***p*-value**	**OR**	**95% CI**	***p*-value**
Age	0.99	0.96–1.02	0.5	1.04	0.99–1.10	0.1
Female*	0.74	0.24–2.27	0.6	–	–	–
BMI	1.01	0.94–1.09	0.8	1.00	0.87–1.15	1.0
Obesity	1.16	0.44–3.05	0.8	0.45	0.06–3.21	0.4
Smoking habit*	1.49	0.59–3.74	0.4	–	–	–
Disease duration	0.99	0.94–1.04	0.7	1.04	0.91–1.20	0.5
Baseline DAS28	1.28	0.96–1.71	0.09	1.14	0.58–2.29	0.7
Baseline CRP	1.01	0.99–1.04	0.3	1.02	0.97–1.08	0.4
Monoclonal TNFi	2.07	0.83–5.12	0.1	0.93	0.15–5.74	0.9
MTX use	1.04	0.44–2.43	0.9	1.60	0.25–10.36	0.6
MTX dose	0.98	0.93–1.02	0.3	1.01	0.92–1.11	0.9
(mg/week)						
Prednisone use	2.46	1.10–5.51	**0.03**	0.75	0.32–9.45	0.5

The univariable logistic regression analysis included 250 patients. Odds ratio (OR) and 95% confidence interval (CI) were calculated. Significant statistical differences are noted in bold. p < 0.05 was considered statistically significant. *Result not provided due to complete separation. BMI, body mass index; DAS28, disease activity score-28; CRP, c-reactive protein; TNFi, tumor necrosis factor inhibitor, MTX, methotrexate.

**Table 4:** Association between patient characteristics and TNFi discontinuation (multivariable analysis), stratified by seropositivity.

	**Seropositive**			**Seronegative**		
**Patients characteristics**	**OR**	**CI 95%**	***p*-value**	**OR**	**IC 95%**	***p*-value**
Age	–	–	–	1.04	0.99–1.10	0.1
Baseline DAS28	1.22	0.90–1.65	0.2	–	–	–
Monoclonal TNFi	2.02	0.78–5.25	0.1	–	–	–
Prednisone use	2.30	1.01–5.23	**0.04**	–	–	–

*Logistic regression analysis adjusted by baseline DAS28 and the dose of concomitant MTX. Odds ratio (OR) and 95% confidence interval (CI) and p-values were calculated. Significant statistical differences are noted in bold. P < 0.05 was considered as statistically significant. DAS28, disease activity score-28; TNFi, tumor necrosis factor inhibitor*.

## Discussion

In this study, we aimed to investigate the effect of using concomitant MTX on the survival of TNFi treatment in patients with RA. In addition, we have analyzed the influence of the patients' characteristics on TNFi discontinuation according to the seropositivity status. In our cohort, we found that the use of concomitant MTX prolonged the median survival time of TNFi in seropositive patients resulting in a 41% smaller likelihood to discontinue TNFi therapy, when compared with patients who did not receive concomitant MTX. In addition, we described that the use of prednisone throughout the TNFi treatment was associated with a higher probability of stopping the therapy.

According to the blood presence of autoantibodies, RA is classified as seropositive or seronegative. The presence of these autoantibodies would confer to the disease a higher degree of inflammation, which results in a more aggressive and joint erosive condition for the patients who show higher disease activity, as well as worse prognosis and an accelerated progression of the disease (5, 6, 19).

Investigations on pharmacokinetics and pharmacodynamics of TNFi have described some factors, which can influence on the survival of these biopharmaceuticals. Sex, disease activity, BMI, the dose and the administration route, the immunogenicity or polymorphisms in Fc receptors, among others, can hamper or increase the treatment survival leading to an increase of its clearance (20). Therefore, the higher disease activity due to the presence of autoantibodies in seropositive patients would be influencing the survival of TNFi treatment (7, 8). We found that seropositive patients from our cohort showed a significantly higher disease activity and a longer disease duration, because they did not respond to previous csDMARDs and needed to initiate their first bDMARD therapy. This would be reflecting the more aggressive and inflammatory disease promoted by the autoantibody presence and therefore, the pivotal role of B cells on the progression of RA (2–4).

It has been demonstrated that the co-administration of csDMARDs, especially MTX, can benefit the TNFi therapy mainly due to a reduction in immunogenicity (12, 21, 22). Herein we found that seropositive patients who received concomitant MTX showed longer TNFi median survival time than those found for patients who were treated without MTX. This result would be in agreement with observations recently published by both Greenwood et al. and van Mulligen et al. (10, 23). In addition, we found that the use of concomitant MTX in seropositive patients resulted in a 41% smaller likelihood of discontinuing TNFi therapy when compared with patients who did not receive it. Nevertheless, the effect of MTX on the B cell is not only restricted to the reduction of development of anti-drug antibodies. MTX would be involved in impairing the development of B cells. As consequence of the mechanism of action of MTX, T cells would reduce the production of cytokines such as TNFα, interleukin-6 (IL6) or Granulocyte macrophage-colony stimulating factor (GM-CSF) (24). IL6 as well as GM-CSF are involved in the development and survival of B cells (25, 26). Consequently, their reduction by MTX may be impairing the development of B cells (27). Furthermore, two studies by our research group reinforced the role of MTX on impairing B cell development. We found that the reduction of peripheral naïve B cells was associated with remission in RA patients who received co-treatment with MTX and TNFi, however, this association was not found in patients who received TNFi monotherapy (28). Moreover, the treatment with MTX was associated with increased serum concentration of B cell activating factor (BAFF) in patients with systemic lupus erythematosus (SLE), which may be due to the diminished uptake by the impaired B cells (29). In addition, low doses of MTX decreased the capacity of B cells to produce autoantibodies such as anti-Ro/SSA and anti-La/SSB in patients with SLE (30). The ability of MTX to decrease the titer of autoantibodies in RA is controversial (31). However, it seems to be documented enough, that moderate-high starting doses of MTX are more effective in autoantibody-seropositive patients than in autoantibody-seronegative patients (32). Nevertheless, more research is needed to demonstrate these hypotheses.

All these data suggest the benefit of the concomitant use of MTX based on the decrease of B cell activity, and consequently the reduction of immunogenicity associated to TNFi therapy, as well as the inflammatory environment. Thus, leading to the TNFi survival in patients with seropositive RA.

Regarding treatment discontinuation, our study showed that seropositive patients who received concomitant prednisone throughout the TNFi therapy had a 2.3 times greater likelihood of discontinuing the treatment. This result was in agreement with previous publications which showed the use of prednisone as a bad predictor of EULAR and ACR70 response, DAS28 and CDAI remission, or drug retention (33, 34). The reason for the negative effect of prednisone on TNFi treatment maintenance is not clear. It appears to be related to the mechanism of the disease (RA), as it does not seem to be associated with TNFi survival in ankylosing spondylitis (35).

One of the strengths of our study was the purity and homogeneity of the population. All the patients included were naïve to biopharmaceutical therapy and starting their first TNFi. Moreover, they had a baseline disease activity by DAS283 > 0.2. Another strength was the limited missing data that has facilitated a robust analysis. Furthermore, discontinuations by improvement or loss of follow-up were not considered. Nevertheless, it can be acknowledge that the analysis of seronegative patients was limited, and can be considered a weakness of the study, due to the small percentage of these patients from our real-data cohort (15%), that represented a population of 37 patients; however, their data were completed collected. Other limitation was that this was a retrospective observational study and the decision of initiating TNFi treatment was made based on the criteria of the treating rheumatologist and therefore, not all the patients followed the same therapeutic strategy before TNFi neither the same criteria to start it. Another limitation was that due the nature of the study bias by indication could not be ruled out and consequently the results needs to be interpreted with caution.

In conclusion, these data provide evidence to support the use of concomitant MTX in seropositive patients in order to prolong the effectiveness and the survival of the TNFi therapy. Moreover, the use of prednisone in seropositive patients receiving TNFi was highly associated with treatment discontinuation.

## Data Availability Statement

The raw data supporting the conclusions of this article will be made available by the authors, without undue reservation.

## Ethics Statement

The studies involving human participants were reviewed and approved by the Institutional Ethics Committee from La Paz University Hospital (PI-1155) in accordance with the Helsinki Declaration. The patients/participants provided their written informed consent to participate in this study. All patients signed an informed consent document before inclusion.

## Author Contributions

BH-B and AB planned the study. BH-B and CB completed the patients' database and performed the statistical analysis. CP-R supervised the statistical analysis. BH-B wrote the manuscript draft. BH-B and AB had full access to all of the data in the study and take responsibility for the integrity of the data and the accuracy of the data analysis. All authors were involved in revising the manuscript critically for important intellectual content, approved the final version to be published, contributed to the manuscript, and approved the submitted version.

## Conflict of Interest

CP-R has received research grants/honoraria from AbbVie, Lilly, Novartis, Pfizer, Sanofi, Biogen and UCB. DP-S reports speaker fees and grants from Abbvie, Grifols, Menarini, Novartis, Pfizer and Takeda during the conduct of the study. AB reports grants, consultancies and speaker fees from Abbvie, BMS, Nordic, Novartis, Pfizer, Sandoz, Sanofi, Roche and UCB during the conduct of the study. The remaining authors declare that the research was conducted in the absence of any commercial or financial relationships that could be construed as a potential conflict of interest.
