# Supplementary material for: Methotrexate Reduces the Probability of Discontinuation of TNF Inhibitors in Seropositive Patients With Rheumatoid Arthritis. A Real-World Data Analysis
Source: Front Med (Lausanne). 2021 Jun 29;8:692557. doi: 10.3389/fmed.2021.692557 (PMC8275858; doi:10.3389/fmed.2021.692557)
Supplement: Supplementary file 1 [file Data_Sheet_1.PDF]

Table S1: Patient characteristics. The table shows mean±SD, median (IQR) or absolute number (percentage) for patients included (n=250). The results are stratified by the use of concomitant MTX. p-value<0.05 was considered statistically significant. BMI, body mass index; DAS28, disease activity score-28; CRP, c-reactive protein; TNFi, tumour necrosis factor inhibitor; RF, rheumatoid factor; ACPA, anti-citrullinated peptide antibody; MTX, methotrexate.

Patient characteristics n=250	No MTX n= 75 (30%)	MTX n= 175 (70%)	p-value
**Age (yr)**	55±15	54±14	0.5
**Female**	60 (80)	148 (85)	0.4
**BMI (kg/m^2)** (n=232)	24.7 (21.7-28.8)	25.8 (22.6-30.3)	0.3
BMI<30 (kg/m^2)	58 (84)	121 (74)	0.1
BMI≥30 (kg/m^2)	11 (16)	42 (26)	0.1
**Smoking habit**			0.3
non-smokers	43 (57)	115 (66)	
ex-smokers	20 (27)	27 (16)	
smokers	12 (16)	32 (18)	
**Seropositivity (RF and/or ACPA)**	67 (89)	146 (83)	0.2
**Disease duration (yr)**	9.5 (3.9-13.1)	7.2 (3.0-12.4)	0.07
**Baseline DAS28**	5.1±1.4	5.2±1.3	0.8
**Baseline CRP (mg/dL)**	8.5 (2.5-18.2)	5.3 (2.6-18.2)	0.5
**TNFi type**			0.6
Infliximab	28 (37)	81 (46)	
Adalimumab	18 (24)	40 (23)	
Etanercept	17 (23)	32 (19)	
Certolizumab	11 (15)	18 (10)	
Golimumab	1 (1)	4 (2)	
**Cause of discontinuation**			0.07
Lack of efficacy	29 (39)	89 (51)	
Other causes	35 (47)	62 (35)	
**Prednisone use**	43 (57)	121 (69)	0.07

Table S2: COX proportional hazard estimates (95% CI) for TNFi therapy survival. Analysis adjusted for age, sex, BMI, smoking habit, disease duration, baseline DAS28, baseline CRP, type of TNFi, cause of TNFi discontinuation, seropositivity and use of prednisone. Significant statistical differences are noted in bold. p-value<0.05 was considered statistically significant. BMI, body mass index; DAS28, disease activity score-28; CRP, c-reactive protein; MTX, methotrexate; TNFi, tumor necrosis factor inhibitor; HR, hazard ratio; CI, confidence interval.

**Patient characteristics**	**HR**	**95% CI**	**p-value**
MTX use (unadjusted)	0.51	0.35-0.74	**0.0004**
MTX use (adjusted)	0.52	0.36-0.74	**0.0003**
Time (year) of TNFi initiation (unadjusted)	1.77	1.48-2.10	**<0.0001**
Time (year) of TNFi initiation (adjusted)	1.76	1.49-2.07	**<0.0001**

Table S3: Association between patient characteristics and TNFi discontinuation (univariable analysis). The univariable logistic regression analysis included 250 patients. Odds ratio (OR) and 95% confidence interval (CI) were calculated. Significant statistical differences are noted in bold. p-value<0.05 was considered statistically significant. *Result not provided due to complete separation. BMI, body mass index; RF, rheumatoid factor; ACPA, anti-citrullinated peptide antibody; DAS28, disease activity score-28; CRP, c-reactive protein; TNFi, tumor necrosis factor inhibitor, MTX, methotrexate.

**Patient characteristics**	**OR**	**95% CI**	**p-value**
**Age**	1.00	0.98-1.03	0.7
**Female**	0.60	0.20-1.80	0.4
**BMI**	1.01	0.94-1.07	0.8
Obesity	1.00	0.42-2.35	1.0
**Smoking habit**	0.56	0.27-1.15	0.1
**Seropositivity (RF and/or ACPA)**	1.54	0.62-3.84	0.3
**Disease duration**	1.00	0.96-1.04	1.0
**Baseline DAS28**	1.28	0.98-1.66	0.06
**Baseline CRP**	1.02	0.99-1.04	0.2
**Monoclonal TNFi**	1.80	0.80-4.06	0.1
**MTX use**	1.08	0.50-2.34	0.8
MTX dose (mg/week)	0.98	0.94-1.02	0.3
**Prednisone use**	2.29	1.11-4.71	**0.02**

Table S4: Association between patient characteristics and TNFi discontinuation (multivariable analysis). Logistic regression analysis adjusted by baseline DAS28 and the dose of concomitant MTX. Odds ratio (OR) and 95% confidence interval (CI) and p-values were calculated. Significant statistical differences are noted in bold. p-value<0.05 was considered as statistically significant. DAS28, disease activity score-28; TNFi, tumor necrosis factor inhibitor.

**Patients characteristics**	**OR**	**CI 95%**	**p-value**
Smoking habit	0.62	0.29-1.33	0.2
Baseline DAS28	1.19	0.90-1.57	0.2
Monoclonal TNFi	1.97	0.83-4.64	0.1
Prednisone use	2.05	0.97-4.32	0.06
